# Factors Associated with the Use of Complementary and Alternative Medicine in Rural Northern Victoria, Australia

**DOI:** 10.1007/s10728-024-00508-9

**Published:** 2025-01-17

**Authors:** Andrew J. Hamilton, Lisa Bourke, Geetha Ranmuthugala, Kristen M. Glenister, David Simmons

**Affiliations:** 1 Department of Rural Health, Melbourne Medical School, University of Melbourne, 49 Graham Street, Shepparton, VIC Australia; 2 Department of Rural Health, University of Melbourne, Docker Street, Wangaratta, VIC Australia; 3 Macarthur Clinical School, Western Sydney University, Locked Bag, Penrith, NSW 1797 Australia; 4 Macarthur Diabetes, Endocrinology and Metabolism Service, Campbelltown Hospital, Campbelltown, NSW 2560 Australia

**Keywords:** Alternative Medicine, Chiropractic, Complementary Medicine, Massage, Osteopathy

## Abstract

About one-third of Australians use the services of complementary and alternative medicine (CAM); but debate about the role of CAM in public healthcare is vociferous. Despite this, the mechanisms driving CAM healthcare choices are not well understood, especially in rural Australia. From 2016 to 2018, 2,679 persons from the Goulburn Valley, northern Victoria, were surveyed, 28% (755) of whom reporting visiting CAM practitioners. A Generalized Linear Mixed Model was used to assess associations between various socio-demographic variables and the use of CAM services. The strongest significant inverse (*p* < 0.05) association with CAM use overall was being unemployed, with markedly lower odds of using CAM than those employed full-time (OR 0.22 [0.12, 0.41]). The next strongest inverse relationship was being retired (OR 0.44 [0.30, 0.65]). The strongest positive associations were with English spoken at home (OR 2.38 [1.34, 4.24]), private health insurance (hospital cover) (1.57 [1.28, 1.91]), being Australian born (OR 1.61 [1.14, 2.28]), and female sex (1.25 [1.02, 1.52])). Females had significantly higher odds of using osteopathy than males (OR 1.98 [1.33, 2.96]) but there were no significant sex differences for chiropractic or massage. This is the first such study conducted solely for a rural Australian population. The drivers of CAM use differed from previous nation-wide studies and they varied across modalities. The factors identified here as being associated with CAM use could be used by CAM practitioners in developing person-centred services. Similarly, the findings are relevant to primary-care services in understanding what sectors of society might eschew conventional health care for CAM in rural regions, where health services are often limited.

## Introduction

Comprising nursing, medicine, dentistry, and allied health, conventional healthcare is the mainstay of the modern public health system. Complementary and alternative medicine (CAM) is outside Australia’s public healthcare system and can be purchased either under some health insurance schemes or through out-of-pocket expenses, operating along a continuum from services that work alongside conventional healthcare to substitutions for it. Debates about the relative efficacies of different CAM treatment approaches are ardent [1–5]. Notwithstanding, CAM offers choice. In Australia, without a medical referral, a patient with a musculoskeletal problem might choose to see an acupuncturist, a chiropractor, a massage therapist, or an osteopath.

Regardless of its formal position in Australian health provision, CAM plays a role and is widely throughout Australia. The most recent economic estimates, 2007, revealed that visits to CAM professionals to be on par with visits to medical professionals (69.2 million and 69.3 million, respectively) [6]. Australians’ out-of-pocket expense on CAM was AU$4.1 billion. A subsequent nation-wide study in 2017 found that 36% of Australians use CAM practitioner services and 63% used either CAM treatments, such as vitamin/mineral supplements, and/or practitioners [6]. Of those who had visited a medical practitioner in the preceding twelve months, 71% had also used CAM practitioner services, with over half (55%) of these not always informing their medical doctors of this. This may suggest a tension or perceived tension between conventional and CAM care, which might, in turn, reflect different socio-demographic profiles of regular or partial CAM users as opposed to those who use conventional medicine alone.

It is crucial to understand the relationships of such socio-demographic factors to use of CAM. It will enable services to be tailored to better meet the needs of the population. Further, it might assist conventional primary healthcare providers in understanding why some people might prefer to use CAM services. Despite this, little is known about these relationships, especially in Australia. When analyzing the effects of demographic factors on use of CAM in Australia at a national level, Steel et al. combined the various individual services into a single variable representing CAM overall [6]. Similarly, another Australian study, restricted to the state of Queensland, treated CAM services collectively [7]. Given the variation in approaches and philosophies behind CAM modalities, it cannot be assumed that demographic factors will operate uniformly across them.

To date, CAM use in an Australian rural setting has received scant attention. CAM use in a part of rural Australia has been addressed in the narrower context of chronic pain [8]. Steel et al.’s (2018) cross-sectional survey was Australia-wide, but analyses were not presented separately for rural and urban regions [6]. Thomson et al. found no difference in overall CAM usage between urban and rural settings in Queensland, and no dedicated analyses were made of the rural data alone [7]. Similarly, von Conrady and Bonney, found no significant effect of rurality on CAM use [9]. A systematic review of Australian CAM studies suggested that rural residents have a stronger affinity for tactile treatments than urban residents [10]. Specifically, use of massage therapists and chiropractors was more common in rural populations, and it has been hypothesized that greater use in rural areas might be a function of poorer access to conventional medicine in these regions [10].

Given the lack of understanding on the use of CAM services in rural Australia, the research question asked here is what the sociodemographic factors are associated with the use of CAM in a rural region of northern Victoria, Australia. Specifically, factors associated with use of chiropractic, massage, osteopathy, and the overall use of CAM were investigated.

## Methods

### Data Collection

Data on the use of CAM were collected as part of the Crossroads-II study in rural Victoria. The study design, scope, randomization procedure, logistics, and rationale for town selection are explained elsewhere [11]. Briefly, from October 2016 to November 2018, 2,679 persons were interviewed at their homes in four Goulburn Valley towns: Benalla (473 persons), Cobram (431), Seymour (431), and Shepparton-Mooroopna (1,344). Demographic data of the sample are placed in the context of the population of the aggregate population across of the four towns in Table 1. The population data were obtained from the 2016 Australian Census, which was the closest census in time to the survey period [12].

### Survey

Participants were asked to identify from lists provided which CAM services they had used within the last twelve months. Only services offered by practitioners were considered; CAM products, such as dietary supplements, were not. The presented list included the following complementary health services: acupuncture, chiropractic, massage therapy, naturopathy, osteopathy. Participants were also given the opportunity to add other services, which saw the addition of applied kinesiology, Bowen therapy, and traditional Chinese medicine. Services were considered to be CAM if they were not part of the Victorian Public Sector enterprise agreement for allied health professionals.

### Analyses

Classifying non-conventional health services into categories such as alternative, traditional, complementary, folk, unorthodox, and natural is fraught with complexity, contradiction, and semantics [13–17]. Here we adopt the term CAM, primarily because it is widely used but also because it includes treatment options that work with conventional medicine and those that are substitutions for it [18]. The practical demarcation used was whether the discipline was *not* included in the Victorian Public Sector enterprise agreement for allied health professionals [19]. This also served to make the distinction apropos to Victoria. The three CAM services used most frequently, chiropractic (376 persons), massage therapy (267 persons), and osteopathy (154 persons) were each analysed independently. Models failed to converge for the modalities used by fewer persons than these. Additionally, the use of any form of CAM was analyzed and named Any CAM.

All binary response variables were modelled with the Binomial distribution, which is the sum of *n* independent Bernoulli random trials. We used Generalized Linear Models (GLM) to do this; specifically, we employed Binomial Generalized Linear Mixed Models (GLMM), which allow for the modelling of a random effect—*town* in this case. *Age*, *sex*,* Year 12 completion as highest educational attainment*, *university completion as highest educational attainment*, *born in Australia*,* private health insurance*,* employment status*, *and English as the primary language spoken at home* were the fixed effects [20]. The logit link function was used for the linear model (i.e., binary logistic regression). An intercept term was included for the fixed effects but not the random effect. In addition to contrasts of each level against the reference level, pairwise *post hoc* comparisons of means were conducted using Fisher’s Least Significant Difference (LSD) test with the acceptable *α* error probability set at 0.05.

Collinearity was tested for using the Variance Inflation Factor (VIF) and variance decomposition. Common bivariate approaches fail to accommodate higher-order correlations [21]. First, auxiliary regressions were conducted and the VIF calculated for each predictor variable [22]. A VIF > 10 was considered to indicate possible collinearity [23–25]. Next, the variances were decomposed, and Condition Indices (η) calculated as a function of the eigenvalue for each singular value (dimension). If, for the row of the *k*^*th*^ singular value of the Π matrix, the Condition Index ($\:{\eta\:}_{k})$ exceeded 15 *and* variance proportions for two or more of the independent variables (save the intercept) exceeded 0.5, then problematic collinearity was deemed to exist [21, 26].

Performance of each GLMM was assessed by determining classification error rates. The cut-off probability for each model was calculated by constructing a Receiver Operating Characteristic (ROC) curve and determining the point on the curve where $\:\text{s}\text{e}\text{n}\text{s}\text{i}\text{t}\text{i}\text{v}\text{i}\text{t}\text{y}\:\left(\text{p}\text{o}\text{w}\text{e}\text{r}\right)\:\left(1-\beta\:\right)=\text{s}\text{p}\text{e}\text{c}\text{i}\text{f}\text{i}\text{c}\text{i}\text{t}\text{y}\:\left(1-\alpha\:\right)$, thus optimizing the trade-off of Type I and II error rates [27]. The Area Under the Curve (AUC) relative to the unit square of 1 was used an indicator of discriminant ability of each GLMM [27].

Statistical analyses were performed using SPSS (Version 29), and the threshold value on the ROC and consequent achieved sensitivities and sensitivities were calculated manually. Unless otherwise stated, significance refers to an α error rate < 0.05.

## Results

28% of participants had used CAM within the previous twelve months (Table 1). The surveyed population comprised mostly females, whereas the sex ratio for the entire population was almost even (Table 1). Over half had completed Year 12 or higher education (Table 1). The mean age of respondents was 54 years (range: 16 to 97 years: CAM users: 16–93; non users: 16–97), which contrasted with a lower median age range, 45–49, for 15 years and above for the population. More spoke English as their primary language at home than was observed for the entire population. Full-time employment frequency was lower and unemployment higher in the sample than observed in the entire population (Table 1). Half (50.2%) of the participants lived in Shepparton-Mooroopna, with the others being spread evenly among the three smaller towns (Table 1).

**Table 1:** Demographics of the surveyed population and the entire population across the four towns. Use/non-use refers to the last 12 months

	Users of CAM	Non-users of CAM	Total sample	Entire population
**Participants**	755	1,924	2,679	71,009
**Age (y)** ^1^	med. 53; mean 51 (17.3)	med. 57; mean 55 (19.8)	med. 56; mean 54 (19.2)	med. 45–49
**Sex**	Female: 461 (61%) Male: 294 (39%)	Female: 1,091 (56%) Male: 833 (43%)	Female: 1,552 (58%) Male: 1,127 (42)	Female: 34,535 (51%) Male: 32,667 (49%)
**Highest education**	< Year 12: 273 (38%) Year 12: 271 (38%) University: 178 (25%)	< Year 12: 801 (44%) Year 12: 662 (36%) University: 362 (20%)	< Year 12: 1,074 (42%) Year 12: 933 (37%) University: 540 (21%)	< Year 12: 23,722 (48%) Year 12: 20,037 (40%) University: 6,162 (12%)
**Language at home**	English: 703 (97%) Other: 19 (3%)	English: 1,651 (92%) Other: 146 (8%)	English: 2,354 (93%) Other: 165 (7%)	English: 52,115 (78%) Other: 15,084 (22
**Town**	Benalla: 135 (18%) Cobram: 112 (15%) Seymour: 94 (12%) Shepp-Moor: 414 (56%)	Benalla: 338 (18%) Cobram: 319 (17%) Seymour: 337 (18%) Shepp-Moor: 930 (48%)	Benalla: 473 (18%) Cobram:431 (16%) Seymour: 431 (16%) Shepp-Moor: 1,344 (50%)	Benalla: 9,298 (14%) Cobram: 5,375 (8%) Seymour: 6,327 (9%) Shepp-Moor: 46,199 (69%)
**Employment**	Full-time: 280 (39%) Part-time/casual: 178 (25%) Unemployed: 162 (22%) Retired: 13 (2%) Other: 89 (12%)	Full-time: 449 (25%) Part-time/casual: 318 (18%) Unemployed: 649 (36%) Retired: 100 (6%) Other: 272 (15%)	Full-time: 729 (29%) Part-time/casual: 496 (20%) Unemployed: 811 (32%) Retired: 113 (5%) Other: 361 (14%)	Full-time: 17,216 (59%) Part-time: 10,378 (36%) Unemployed: 1,543 (5%) ^2^Retired: NA ^2^Other: NA

^1^The value in parentheses after the mean is the standard deviation of the mean. For the population, the estimate is the weighted median age category across the four towns and starting from the closest ABS age category, 15–19 years, to the minimum age in the current study (16 years). Single-year resolution data were not available. ^2^No data for retired and other categories

There was no significant relationship between age with use of chiropractic, massage, osteopathy, or Any CAM (Tables 2 and 3) Females had a significantly greater relationship with use of osteopathy (*p* = < 0.001, OR 1.98 [1.33, 2.96]) and Any CAM (*p* = 0.030, OR 1.25 [1.02, 1.52]) than males, but there were no such significant sex differences for chiropractic (*p* = 0.519, OR 1.09 [0.85, 1.40]) or massage (*p* = 0.449, OR 1.12 [0.84, 1.49]). Persons who held Year 12 as their highest level of education had significantly lower odds of using chiropractic (OR 0.70 [0.53, 0.93], *p* = 0.015), but no such significant differences were found for the other CAM modalities, including Any CAM (Tables 2 and 3). For those with university as their highest qualification, higher odds relative to no Year 12 were observed for chiropractic (*p* = 0.001, OR 0.56 [0.40, 0.79]) and lower odds for osteopathy (*p* = 0.033, OR 1.68 [1.04, 2.72]]).

Private health insurance hospital cover was significantly associated with greater odds of the use of chiropractic (*p* < 0.001, OR 1.80 [1.39, 2.31]), osteopathy (*p* < 0.001, OR 2.38 [1.61, 3.53]), and Any CAM (*p* < 0.001, OR 1.57 [1.28, 1.91]) but not with massage (*p* = 0.924, OR 1.01 [0.76, 1.36]). In comparison to full-time (FT) employment, there were significantly lower odds of using chiropractic if retired (*p* < 0.001, OR 0.40 [0.27, 0.62]) or unemployed (*p* = 0.017, OR 0.43 [0.22, 0.86], ), but there was no difference for part-time/casual employees (*p* = 0.263, OR 0.83 [0.60, 1.15]). Similarly, the odds of the unemployed using massage therapy were significantly lower than for those employed FT (*p* = 0.002, OR 0.24 [0.09, 0.67], ), as were the odds for the retired (*p* = 0.002, OR 0.48 [0.30, 0.77]). The unemployed and the retired had greater odds of using osteopathy (unemployed: *p* = 0.027, OR 0.20 [0.05, 0.84]; retired: *p* < 0.001, OR 0.21 [0.11, 0.41]). The odds of using Any CAM were relative to the reference level of full-time were significantly lower for the retired (*p* < 0.001, OR 0.35 OR [0.25, 0.48]) and the unemployed (*p* < 0.001, OR 0.22 [0.12, 0.40]). Placing aside contrasts against the reference level and with the ‘other’ category, the only significant pairwise difference for massage was between PT/casual and unemployed. All contrasts were significantly different from each other for Any CAM, save retired versus unemployed. For chiropractic and osteopathy, significant differences were observed between unemployed and PT/casual and unemployed and retired.

**Table 2:** Socio-demographic factors and use of chiropractic and massage

Variable	Chiropractic (*n* = 376)		Massage (*n* = 267)	
	OR [CI_95_]	*p*	OR [CI_95_]	*p*
**Age (Years)**	1.00 [0.99, 1.01]	0.564	0.99 [0.98, 1.00]	0.204
**Sex (Male)**	—	—	—	—
Female	1.09 [0.85, 1.40]	0.519	1.12 [0.84, 1.49]	0.449
**Born in Australia (No)**	—	—	—	—
Yes	1.22 [0.78, 1.89]	0.383	1.72 [1.00, 2.97]	0.051
**Education (No Year 12)**	—	0.002	—	0.283
Year 12	0.70 [0.53, 0.93]	0.015	0.98 [0.70, 1.36]	0.894
Bachelor	0.56 [0.40, 0.79]	0.001	1.29 [0.88, 1.87]	0.191
**Private insurance (No)**	—	—	—	—
Yes	1.80 [1.39, 2.31]	< 0.001	1.01 [0.76, 1.36]	0.924
**Employment (FT)**	—	< 0.001	—	0.001
Part-time/casual	0.83 [0.60, 1.15]	0.263	0.89 [0.62, 1.27]	0.505
Retired	0.40 [0.27, 0.62]	< 0.001	0.48 [0.30, 0.77]	0.002
Unemployed	0.43 [0.22, 0.86]	0.017	0.24 [0.09, 0.67]	0.007
Other	0.54 [0.36, 0.81]	0.003	0.58 [0.37, 0.91]	0.017
**English at home (No)**	—	—	—	—
Yes	2.66 [1.18, 6.02]	0.019	1.59 [0.69, 3.63]	0.275
**Intercept**	0.09 [0.03, 0.21]	< 0.001	0.08 [0.03, 0.20]	< 0.001

The reference category is denoted in parentheses, and the *p*-values given in the first row for age and employment relate to the overall association for the variable

**Table 3:** Socio-demographic factors and use of osteopathy and any CAM. Presentation as per table 2

Variable	Osteopathy (*n* = 154)		Any CAM (*n* = 1,036)	
	OR [CI_95_]	*p*	OR [CI_95_]	*p*
**Age (Years)**	1.00 [0.99, 1.01]	0.850	1.00 [0.99, 1.01]	0.946
**Sex (Male)**	—	—	—	—
Female	1.98 [1.33, 2.96]	< 0.001	1.25 [1.02, 1.52]	0.030
**Born in Australia (No)**	—	—	—	—
Yes	3.793[1.36, 10.59]	0.011	1.61 [1.14, 2.28]	0.007
**Education (No Year 12)**	—	0.055	—	0.627
Year 12	1.07 [0.68, 1.71]	0.764	0.90 [0.72, 1.13]	0.374
Bachelor	1.68 [1.04, 2.72]	0.033	0.99 [0.76, 1.29]	0.941
**Private insurance (No)**	—	—	—	—
Yes	2.38 [1.61, 3.53]	< 0.001	1.57 [1.28, 1.91]	< 0.001
**Employment (FT)**	—	< 0.001	—	< 0.001
Part-time/casual	0.50 [0.32, 0.80]	0.004	0.79 [0.61, 1.02]	0.068
Retired	0.21 [0.11, 0.41]	< 0.001	0.35 [0.25, 0.48]	< 0.001
Unemployed	0.20 [0.05, 0.84]	0.027	0.22 [0.12, 0.40]	< 0.001
Other	0.61 [0.34, 1.08]	0.092	0.53 [0.39, 0.72]	< 0.001
**English at home (No)**	—	—	—	—
Yes	2.55 [0.51, 12.71]	0.253	2.38 [1.34, 4.24]	0.003
**Intercept**	0.01 [0.00, 0.02]	< 0.001	0.14 [0.08, 0.27]	< 0.001

The reference category is denoted in parentheses, and the *p*-values given in the first row for age and employment relate to the overall association for the variable

The random effect, *town*, was insignificant for all models (*p* > 0.3 for all). All fixed effects models were a significant improvement (*p* < 0.001) on the intercept-only model. The performance of the models varied (Table 4). The osteopathy model had the best discriminatory ability and was the most accurate at classifying, and there was little variation among the others on these dimensions (Table 4). Sensitivity and specificity were well balanced across all models. Nonetheless, the osteopathy model was the only one for which sensitivity exceeded specificity, but the difference was only 0.9%. The differentials were larger for the other models and in favour of specificity, suggesting that they were slightly better at classifying no use of a CAM service when, in fact, the service was not used, and poorer at predicting use when it truly did occur.

**Table 4:** Classification performance of the models

GLMM	AUC [CI_95_]	Specificity (%)	Sensitivity (%)	Accuracy (%)
Chiropractic	0.660 [0.629, 0.690]	58.7	65.4	59.6
Massage	0.650 [0.615, 0.686]	63.5	59.2	63.1
Osteopathy	0.769 [0.731, 0.807]	71.6	71.2	71.5
Any CAM	0.670 [0.646, 0.693]	62.2	62.3	62.2

## Discussion

This study investigated the socio-demographic factors associated with the use of CAM services. Being unemployed had the strongest association with the odds of using CAM, decreasing the odds of use markedly, followed by being retired. This may relate to restrictions in income. The factor that increased the odds the most was speaking English at home. Similar trends were observed across the different modalities analyzed, with the most notable exception being a large increase in odds of using osteopathy by those born in Australia.

The frequency of use of CAM observed here, 28%, was markedly lower than that reported in a recent nation-wide Australian study (36% CAM practitioner) and another in the state of Queensland (79%: practitioner and/or self-care) [6, 7]. This escapes ready explanation. It might be due to the fact that these other studies are not exclusively rural, and in fact are overwhelming urban, with neither separating urban and rural in their interpretations of the results. It can be posited that rural populations in northern Victoria are less well serviced with CAM practitioners. There might be cultural differences with urban areas and/or population densities below critical mass in some towns to support a particular CAM therapist, resulting in the need for travel to a larger town. The only relationship observed here between use of a CAM modality and education level was the positive relationship with Year 12 completion for chiropractic. The reason no education relationships were observed for the other modalities and Any CAM is unclear. Placing aside debates about efficacy, chiropractic purports to offer a holistic approach of body systems, but so does osteopathy and, to a degree, massage [28–30]. Previous Australian studies have noted education effects on CAM use, but CAM was not disaggregated [6, 31]. Similarly, an increase in use of CAM with higher education level has been observed, but these studies did not analyze the various services individually [6, 31].

The negative associations with not speaking English at home and overall CAM use and use of chiropractic might reflect multifaceted associations with being an immigrant. Such factors were not available here but have been addressed in other contexts. An analysis of the United States’ National Health Interview Survey (*n* = 34,483) found that use of CAM tends to decrease from those born in the US to naturalized citizens to non-citizen immigrants [32]. Naturalized citizens had significantly lower odds than US-born citizens of using biologically based therapies and manipulative and body-based therapies, and the odds for both lower yet again for non-citizens. Further, non-citizens had significantly lower odds than US-born citizens of using mind-body therapies. Various factors were allied with accessibility, cost, and knowledge/awareness differed between these immigrant categories and US-born and were suggested to be possible drivers behind the observed discrepancies in use of these CAM services. The use of CAM by immigrants in Australia is poorly understood, and factors associated with use have received negligible attention, and virtually no attention rural Australia. Here, language had a negative association with CAM use in a part of regional Australia, and it might be indicative of associations related to experiences of immigrants.

Here, women had greater odds of using osteopathy and Any CAM but not chiropractic or massage than men. Some studies have revealed a female preference for CAM more broadly [32, 33], but associations with specific factors are yet to be determined. Further, women have higher rates of chronic pain than men, and, in a broader context, they have been observed to avail themselves of healthcare services more frequently and earlier [33–35].

The private health insurance variable used here was for hospital cover. Many insurance schemes attach extras to hospital cover, with these often including some CAM services. Further, given no information on income was available, we included private health insurance as a potential proxy for means. This is supported by an Australian Bureau of Statistics study which found 34% of those in the first quintile of socioeconomic disadvantage (i.e., the most disadvantaged) had private health insurance compared to 80% in the fifth (least disadvantaged) quintile, with the intermediate quintiles following a linear trend between these two extremes [36]. The greater odds of Any CAM use by those with private insurance, as well as greater odds of use of osteopathy and chiropractic, but not massage, might result from either cover of these services by the insurance schemes or from greater wealth of those using them, or both. Through their hospital extras packages, at the time of the survey three of Australia’s largest health insurance schemes all offered substantial cover of chiropractic and osteopathy but only one covered massage therapy [37–39].

No age relationships with chiropractic, massage, osteopathy, or Any CAM were found. In contrast Drivdahl and Miser presented a middling effect, with higher use of CAM for middle age groups, but no probabilistic tests were made between the levels [40]. Adams et al. found that Australian women in the middle-age group were more likely to use CAM than those in the younger or older groups [41]. In the middle-age category 39% of CAM users reported having gone through menopause in the previous year [41].

Relationships with employment status and age tended to vary across the different CAM modalities. The practice of assessing predictors against CAM collectively risks missing determinants for specific CAM services [6–9, 40, 42, 43]. On the other hand, a collective category can be useful for capturing the many less popular CAM modalities, as was revealed here with no sex effect emerging for any of the five main CAM treatments but a significant effect for Any CAM. This study was limited geographically to a region of northern Victoria, and extrapolations would need to be made with care. It was also conducted prior to the COVID-19 pandemic. It also lacked data on health literacy and access more broadly, which have been found to be relevant in other Australian settings [6, 10, 44].

In conclusion, CAM is a diverse system of healthcare, and efforts to understand how and why the numerous modalities are used demands accommodation of such variation. This is particularly important given the contested dialogue on the efficacies of CAM treatments. Here we found that being employed was strongly associated with using CAM services, as was having English as one’s mother tongue. Further research is required to determine why such associations exist. While CAM sits outside Australia’s formal healthcare system, it is an option that should be equally available. It is thus crucial that those without employment and those with language barriers are not excluded from such services. Trends were similar across CAM modalities, with the clear exception of osteopathy, which saw greater use by those born in Australia. Many CAM services were used by insufficient numbers of people to facilitate disaggregated analysis, and thus were represented in the overall Any CAM alone. Future studies with larger sample sizes would be required to assess directly associations with the lesser-used CAM modalities. More broadly, this study was unique in relating solely to a rural region. Given the socio-demographic variation across rural Australia, further studies in other rural areas are needed for drawing broader inference for rural Australia.
